# Bromide of Ethyl

**Published:** 1880-06

**Authors:** 


### EXCERPTA.

Bromide of Ethyl.—This new anæsthetic is continuing to receive a great deal of attention, and the indications are that it may yet be relieved of the suspicion cast on it by the death in one case, and alarming symptoms in others, which have been reported as having followed its employment. There is only one way of reconciling the long run of successes in the hands of Dr. Levis with the decidedly dangerous symptoms developed in the hands of the majority of those who have reported the results of their trials of it more recently, and that is on the supposition that the preparation employed by Dr. Levis was pure, while that employed by the others was adulterated with deleterious ingredients. That impure preparations have been put on the market admits of no doubt, and to hold bromide of ethyl responsible for these is scarcely just, but it is eminently proper to enquire whether the impurities are capable of giving rise to the symptoms reported. An analysis of several preparations by Prof. Jungk, of the Michigan College of Medicine, of this city, reveals the presence of these impurities, and the experiments he reports in the *Therapeutic Gazette* forces one to the conclusion that they are capable of the disturbances recently noted in the use of the bromide of ethyl. Whether they are the sole cause of these disturbances remains yet to be determined.

In the discussion following the report of the fatal case by Dr. Sims to the New York Medical Society, Dr. Squibb undertook to account for the poisonous effects of bromide of ethyl by assuming it to be a loosely molecular article, easily decomposed, that thus its administration is prone to be followed by an impregnation of the system with bromine, and that if it remained as bromide of ethyl in the system it might not be harmful. This theory has been shown to be based on insufficient grounds. In the first place, as Prof. Jungk has shown, bromide is not "a loosely molecular article," that in fact it is a very stable salt (for a salt it really is), and very difficult of decomposition, much more difficult than chloroform. In the second place, the assumption that anæsthesia is due to a breaking up of the anæsthetic into its elements is nothing more than a hypothesis, and one, too, which has little or nothing to support it. The fact that it is one of the characteristics of bromide of ethyl that it is perfectly unirritating to the bronchi goes to show that it is not decomposed; if it were, the bromine in its composition, one of the most irritant of substances, would certainly manifest itself in its effects on the air passages.

As the matter now stands, it is evident that it was an impure preparation of bromide of ethyl which produced the unfavorable results in each case thus far reported. This fact is very unfortunate—unfortunate not only because of the results thus far, but because of the suspicion it casts on the manufacturers. Manufacturing pharmacists have of late been severely criticized in some quarters, and if it shall appear that they have, either through ignorance, carelesness or design, placed a deadly preparation of an innocuous article in the hands of the profession, the shaken confidence must be still further impaired. We await developments with much interest.—*Michigan Medical News.*

